# Spinal Injury Associated With Firearm Use

**DOI:** 10.7759/cureus.13918

**Published:** 2021-03-16

**Authors:** Randall T Loder, Abhipri Mishra, Bradley Atoa, Allison Young

**Affiliations:** 1 Orthopaedic Surgery, Riley Hospital for Children, Indianapolis, USA; 2 Orthopaedic Surgery, Indiana University School of Medicine, Indianapolis, USA

**Keywords:** firearm, injury, spine, neurologic injury, cervical, thoracic, lumbar, demographics

## Abstract

Objective

Injuries associated with firearms are a significant health burden. However, there is no comprehensive study of firearm spinal injuries over a large population. It was the purpose of this study to analyze the demographics of spinal firearm injuries across the entire United States for all ages using a national database.

Methods

A retrospective review of prospectively collected data using the Inter-University Consortium for Political and Social Research Firearm Injury Surveillance Study 1993-2015 (ICPSR 37276) was performed. The demographic variables of patients with spinal injuries due to firearms were analyzed with statistical analyses accounting for the weighted, stratified nature of the data, using SUDAAN 11.0.01™ software (RTI International, Research Triangle Park, North Carolina, 2013). A p-value of < 0.05 was considered statistically significant.

Results

For the years 1993 through 2015, there were an estimated 2,667,896 emergency department (ED) visits for injuries due to firearms; 10,296 of these injuries (0.4%) involved the spine. The vast majority (98.2%) were due to powder firearm gunshot wounds. Those with a spine injury were more likely to have been injured in an assault (83.7% vs. 60.2%), involved a handgun (83.5% vs. 60.2%), were male (90.8% vs. 86.4%), were admitted to the hospital (86.8% vs. 30.9%), and were seen in urban hospitals (86.7 vs. 64.6%). The average age was 28 years with very few on those < 14 years of age. Illicit drug involvement was over four times as frequent in those with a spine injury (34.7% vs. 8.0%). The cervical spine was involved in 30%, thoracic in 32%, lumbar in 32%, and sacrum in 6%. A fracture occurred in 91.8% and neurologic injury in 33%. Injuries to the thoracic spine had the highest percentage of neurologic involvement (50.4%). There was an annual percentage decrease for patients with and without spine involvement in the 1990s, followed by increases through 2015. The average percentage increase for patients with a spine injury was 10.3% per year from 1997 onwards (p < 10^-6^), significantly greater than the 1.5% for those without spinal involvement (p = 0.0001) from 1999 onwards.

Conclusions

This nation-wide study of spinal injuries associated with firearms covering all ages can be used as baseline data for future firearm studies. A reduction in the incidence of such injuries can be guided by our findings but may be difficult due to sociopolitical barriers (e.g. socioeconomic status of the injured patients, differences in political opinion regarding gun control in the US, and geospatial patterns of firearm injury).

## Introduction

Injuries associated with firearms are a significant health burden [1-3]. While firearm injuries represent only 4% of injuries seen at major trauma centers (National Trauma Databank information), deaths attributed to firearms in the population are equivalent to those from motor vehicle crashes and falls [2]. They also result in significant costs to society, both financially and in loss of human life/work [3-5]. Firearm injuries account for more than an annual $70 billion in costs [3] to the US health care system. Ranney [4] noted that in the six months after a firearm injury, patient-level health care visits and costs increased three to 20 times when compared to the six months prior. They also account for the sizeable human loss of life [5]; for those with a gunshot spinal cord injury due, the life expectancy loss for each person with quadriplegia is 17 years and with paraplegia 11.4 years. This equates to 25,647 years of life lost each year due to new spinal cord gunshot injuries.

There is some literature regarding firearm injuries to the spine, however many focus on only one anatomic area (e.g., cervical), multiple mechanisms of injury, including firearms, specific age groups, war injuries, general reviews regarding treatment, or case reports. Furthermore, there are no comprehensive studies of firearm spinal injuries over a large population. The aim of this study was to analyze injuries to the spine due to firearm activity across the entire United States for all ages using a national database. Such data will be useful as baseline data for future studies regarding spinal injuries due to firearms and can serve as a guide for injury prevention programs. This also begins to fill a void in the paucity of firearm research, which has been recently noted [6].

## Materials and methods

The data for this study were obtained from the Inter-University Consortium for Political and Social Research Firearm Injury Surveillance Study 1993-2015 (ICPSR 37276) [7] collected by the National Electronic Injury Surveillance System (NEISS). The NEISS, a branch of the US Consumer Product Safety Commission, collects data from a probability sample of hospitals in the United States and its territories that have at least six beds and an emergency department (ED). The sample contains five strata: four based on size (the total number of emergency room visits reported by the hospital and are small, medium, large, and very large) and one consisting of children’s hospitals. The NEISS is composed of ~100 hospitals, as this number varies slightly from year to year. Patient information is collected daily from each NEISS hospital for every patient treated in the ED due to an injury associated with consumer products. For this particular study, the ICPSR data set consists of any patient seeking care in the ED for any firearm-related injury, regardless of activity involved during the injury (e.g. hunting, committing a crime, suicide, assault), and whether or not the patient sustained a gunshot wound (coded as GSW by NEISS) or injured in some other way (coded as NGSW by NEISS). Examples of an NGSW are a laceration while cleaning a firearm, head trauma from being pistol-whipped, a clavicle fracture from a rifle recoil, etc. Further details regarding the acquisition of ICPSR/NEISS data and guidelines for use of such data can be accessed from their respective websites (ICPSR - www.icpsr.umich.edu, NEISS -www.cpsc.gov/library/neiss.html). 

The data for 1993 through 2015 due to firearms were downloaded from the ICPSR website. This data set includes age/age groups, injury diagnosis, gender, race, marital status, type of firearm, the perpetrator of injury, intent of injury (unintentional, assault, suicide, law enforcement), anatomic location of the injury, method of transportation to the ED, disposition from the ED, the involvement of drugs/crimes/fights/arguments in the incident, and whether or not the patient was shot. The race was classified as White, Black, Amerindian (Hispanic and Native American), and Indo-Malay (Asian origin) [8]. This study was considered exempt by our local institutional review board.

Injuries involving the spine were ascertained by reviewing the cases and narrative comments for those with a BDYPT (body part) code of 31 (upper trunk), 79 (lower trunk), and 89 (neck) and using the diagnosis codes of fracture (57), internal organ injury (62), and nerve damage (61). Next, all the narrative comments were searched using the FIND command in Microsoft Excel™ (Microsoft® Office 365, Microsoft Corporation, Redmond, WA)) using the keywords: vert, sacr, cocc, thor, lumbar, cerv, atlas, axis, quad, para, as well as each individual vertebra (ie. C1, 2, . . . , L5). A neurologic injury was considered present when the diagnosis code was 61 (nerve damage) and/or when the search of the narrative comments was positive for paraplegia, quadriplegia, or paralyzed/paralysis and when the diagnosis code 62 (internal organ injury) was associated with a neurologic injury in the narrative comments. The NEISS does not report an American Spinal Injury Association Impairment Scale or Injury Severity Score.

We also wished to analyze the prevalence of sexual assault and alcohol involvement with these events. Sexual assault was determined by searching for the keywords of rape, sex, sexual assault, incest, sodomy, intercourse, ejaculate, penetration, vagin, oral, and anal. Alcohol involvement was determined by searching for the keywords alcohol, EtOH, intoxicated, drinking, drank, drunk, club, ethanol, saloon, tavern, liquor, booze, beer, whiskey, brandy, rum, vodka, scotch, tequila, wine, sake, champagne, cognac, and BAC (an acronym for blood alcohol involvement).

Statistical analysis

Statistical analyses were performed with SUDAAN 11.0.01™ software (RTI International, Research Triangle Park, North Carolina, 2013) to account for the stratified and weighted nature of the data. The estimated number of ED visits was calculated, along with 95% confidence intervals (CIs) of the estimate. (Throughout the remainder of the manuscript when numbers are denoted as {x, y}, these represent the 95% CIs of the estimate). When the actual number of patients (n) is < 20, the estimated number (N) becomes unstable and should be interpreted with caution; thus both n and N were reported. Analyses between groups of continuous data were performed with the t-test (two groups) or analysis of variance (ANOVA) (three or more groups). Differences between groups of categorical data were analyzed by the c2 test. Joinpoint regression analysis was used to analyze for percentage changes over time (Joinpoint Regression Program, Version 4.8.0.1, April 2020; Statistical Research and Applications Branch, National Cancer Institute [https://surveillance.cancer.gov/joinpoint/]). For all analyses, a p < 0.05 was considered statistically significant.

## Results

Analyses between patients and without a spine injury

**Figure 1:**
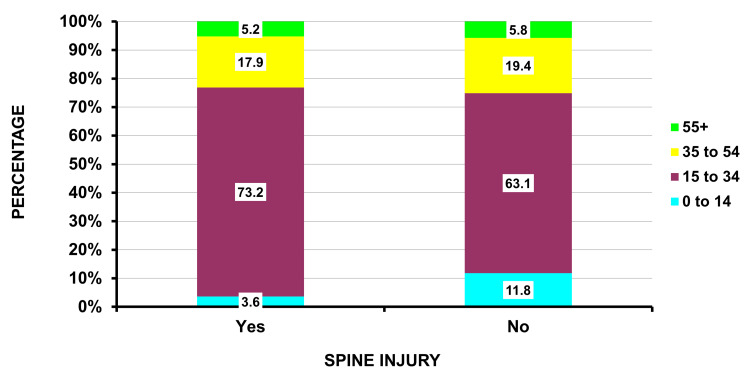
Age groupings for those with and without spine injury due to firearms (p = 0.0001) The actual percentages are shown in each cell.

Patients with a spine injury (Table 1) were more likely to have been injured during an assault (83.7% vs. 60.2%; p = 0.0009), involved a handgun (83.5% vs. 60.2%; p = 0.0001), male sex (90.8% vs. 86.4%; p = 0.003), admitted to the hospital (86.8% vs. 30.9%; p = 0.0006), and seen in larger hospitals (86.7 vs. 64.6%; p = 0.006). The injury was less commonly self-inflicted (9.9% vs. 25.9%; p = 0.028). Although there was no overall difference in the average age between the patients with a spinal injury compared to those without (28.1 vs. 27.8 years; p = 0.67), there was a marked difference when broken down by age groups, with very few spinal injuries in patients < 14 years of age (Figure 1). Illicit drug involvement was over four times as frequent in patients with a spine injury (34.7% vs. 8.0%; p = 0.0052), and involvement in a crime was 1.5 times as frequent (40.6% vs. 27.6%; p = 0.046) in patients with a spine injury. There were no sexual assaults in the spinal injury group.

**Table 1:** Demographics of those with and without a spine injury and firearm use n = actual number of ED visits, N = estimated number of ED visits, L95% = lower 95% CI, U95% = upper 95% CI ED: emergency department

	Spine involvement					No spine involvement					
	n	N	L95%	U95%	%	n	N	L95%	U95%	%	p value
All	420	10,296	7,205	14,944	0.4	90,720	2,658,361	2,653,713	2,661,452	99.6	
Age (years)											
Mean [95% CI]	28.1 [26.6, 29.5]					27.8 [27.1, 28.4]					0.67
Median [interquartile]	23.5 [19.3, 33.2]					23.5 [17.7, 34.1]					
Injury intent											
Unintentional	22	776	426	1,372	7.9	19,998	790,532	638,015	959,658	33.0	0.0009
Assault	349	8,192	7,433	8,742	83.7	56,317	1,441,298	1,259,506	1,612,043	60.2	
Suicide	18	580	318	1,035	5.9	3,774	131,815	95,559	180,579	5.5	
Law enforcement	11	241	144	401	2.5	932	31,308	22,992	42,630	1.3	
Firearm type											
Handgun	104	2,617	2,278	2,839	83.5	24,781	701,369	587,132	816,930	48.3	0.0001
Rifle	8	287	145	541	9.2	3,796	145,263	110,105	189,997	10.0	
Shotgun	5	224	60	732	7.1	3,256	131,436	112,865	152,666	9.0	
BB	1	6	1	47	0.2	12,680	474,511	391,615	565,634	32.7	
Hospital size											
Small	7	589	228	1,441	5.7	6,476	507,349	363,664	690,642	19.1	0.0061
Medium	15	690	257	1,727	6.7	7,430	406,425	275,406	584,308	15.3	
Large	69	3,988	1,511	7,198	38.7	13,608	758,313	404,071	1,251,025	28.5	
Very large	313	4,937	2,426	7,553	48.0	58,525	958,628	643,589	1,326,522	36.1	
Children's	16	92	38	222	0.9	4,681	27,646	18,077	42,534	1.0	
Sex											
Male	379	9,332	8,950	9,612	90.8	78,802	2,295,005	2,267,481	2,320,634	86.4	0.0033
Female	39	944	664	1,326	9.2	11,888	362,615	336,986	390,139	13.6	
Race											
White	80	2,830	2,241	3,481	35.1	23,843	931,455	740,477	1,133,254	42.6	0.059
Black	146	3,053	1,803	4,540	37.9	38,409	872,340	626,255	1,144,195	39.9	
Amerindian	64	2,072	1,193	3,287	25.7	9,368	363,148	203,062	610,719	16.6	
Asian	6	98	35	271	1.2	866	21,228	11,378	39,606	1.0	
Incident locale											
Home	68	1,969	1,510	2,461	41.6	20,327	732,937	631,178	836,432	47.1	0.011
School/recreation	14	354	153	775	7.5	1,986	78,502	64,005	96,242	5.0	
Street/highway	53	1,265	848	1,792	26.7	18,310	434,890	316,601	576,828	27.9	
Other property	47	1,144	834	1,523	24.2	10,250	304,040	252,129	363,632	19.5	
Farm	0	0	0	0	0.0	127	6,942	4,360	11,057	0.4	
Transportation to ED											
Emergency medical service	349	8,414	7,860	8,843	84.4	49,921	1,255,959	1,049,318	1,458,588	52.8	0.0001
Air	27	808	473	1,346	8.1	1,917	58,535	34,462	98,396	2.5	
Private vehicle	13	521	272	976	5.2	17,930	698,677	545,218	874,630	29.4	
Walk-in	5	144	64	321	1.4	8,161	283,043	203,446	388,355	11.9	
Police	5	79	15	418	0.8	2,977	72,219	35,413	145,217	3.0	
Other	0	0	0	0	0.0	237	8,278	4,040	16,399	0.3	
Anatomic location of injury											
Head/neck	126	3,015	3,593	306,716	29.6	25,521	793,383	740,891	847,663	30.5	0.0004
Upper trunk	138	3,605	4,305	366,737	35.4	14,266	381,696	334,378	434,639	14.7	
Lower trunk	148	3,427	3,894	348,629	33.7	11,123	289,804	257,815	325,003	11.1	
Upper extremity	1	82	610	8,342	0.8	14,783	491,967	442,972	545,057	18.9	
Lower extremity	4	44	137	4,476	0.4	23,075	647,338	615,109	680,735	24.9	
Diagnosis											
Contusion/abrasion	0	0	0	0	0.0	5,069	166,538	142,625	194,201	6.3	<10-4
Foreign body	23	576	1,222	59,305	5.6	9,135	323,836	248,409	417,875	12.3	
Fracture	163	3,752	4,747	386,306	36.4	6,523	188,859	157,624	225,779	7.2	
Laceration	34	839	1,410	86,383	8.1	9,807	335,217	263,935	422,348	12.7	
Internal organ injury	61	1,093	2,413	112,535	10.6	4,640	127,629	99,469	163,413	4.9	
Puncture	54	2,974	4,282	306,203	28.9	28,777	840,801	671,810	1,029,951	32.0	
Not stated	45	1,062	1,964	109,344	10.3	26,026	648,574	497,345	827,855	24.6	
ED Disposition											
Treated and released	35	1,309	793	2,086	12.8	51,326	1,686,619	1,503,591	1,855,563	64.0	0.0006
Admit	382	8,873	8,097	9,394	86.8	33,742	813,584	664,132	979,984	30.9	
Fatal	2	37	9	145	0.4	4,910	136,289	115,742	160,299	5.2	
Marital Status											
Never married	150	4,017	3,488	4,442	74.0	33,207	927,325	810,059	1,029,794	68.0	0.20
Married	26	910	585	1,364	16.8	7,619	308,953	244,696	384,366	22.7	
Divorced/separated	7	173	96	308	3.2	1,564	60,117	44,329	81,156	4.4	
Other	6	327	87	1,093	6.0	1,239	67,661	24,961	173,906	5.0	
Argument											
Yes	30	515	193	1,126	19.6	5,440	165,558	136,973	198,871	15.0	0.54
No	69	2,115	1,504	2,437	80.4	25,064	941,741	908,428	970,326	85.0	
Crime											
Yes	61	1,319	908	1,775	40.6	15,238	352,085	230,102	507,398	27.6	0.046
No	56	1,926	1,470	2,337	59.4	13,695	923,426	768,113	1,045,409	72.4	
Illicit drug involvement											
Yes	35	958	709	1,242	34.7	2,457	83,146	46,498	144,475	8.0	0.0052
No	60	1,804	1,520	2,053	65.3	25,781	954,749	893,420	991,397	92.0	
Fight											
Yes	36	872	555	1,291	25.1	7,768	229,151	189,007	275,510	19.0	0.20
No	83	2,609	2,190	2,926	74.9	25,879	973,951	927,592	1,014,095	81.0	
Alcohol involvement											
Yes	29	818	521	1,261	7.9	4,081	143,694	87,538	236,836	5.4	0.056
No	391	9,478	9,035	9,775	92.1	86,636	2,514,519	2,448,377	2,597,675	93.6	
Sexual assault											
Yes	0	0	0	0	0.0	505	13,067	2,640,018	2,649,057	0.5	0.0012
No	420	10,296	7,205	14,944	100.0	90,214	2,645,278	0	0	99.5	
Who caused											
Unknown	260	5,773	4,879	6,578	56.3	44,887	1,103,518	971,206	1,227,885	41.7	0.028
Stranger	54	1,400	1,083	1,776	13.7	13,311	369,553	314,307	428,240	14.0	
Self	30	1,017	627	1,596	9.9	17,077	683,502	558,034	817,357	25.9	
Friend/acquaintance	19	520	281	935	5.1	5,425	180,846	152,792	211,476	6.8	
Spouse/ex	2	59	12	281	0.6	522	19,137	15,332	23,527	0.7	
Other relative	5	135	60	294	1.3	2,065	76,853	61,593	94,636	2.9	
Other/not seen	50	1,392	1,008	1,875	13.6	7,433	224,952	184,778	270,161	8.5	

	Spine involvement					No spine involvement					
	n	N	L95%	U95%	%	n	N	L95%	U95%	%	p value
All	420	10,296	7,205	14,944	0.4	90,720	2,658,361	2,653,713	2,661,452	99.6	
Age (years)											
Mean [95% CI]	28.1 [26.6, 29.5]					27.8 [27.1, 28.4]					0.67
Median [interquartile]	23.5 [19.3, 33.2]					23.5 [17.7, 34.1]					
Injury intent											
Unintentional	22	776	426	1,372	7.9	19,998	790,532	638,015	959,658	33.0	0.0009
Assault	349	8,192	7,433	8,742	83.7	56,317	1,441,298	1,259,506	1,612,043	60.2	
Suicide	18	580	318	1,035	5.9	3,774	131,815	95,559	180,579	5.5	
Law enforcement	11	241	144	401	2.5	932	31,308	22,992	42,630	1.3	
Firearm type											
Handgun	104	2,617	2,278	2,839	83.5	24,781	701,369	587,132	816,930	48.3	0.0001
Rifle	8	287	145	541	9.2	3,796	145,263	110,105	189,997	10.0	
Shotgun	5	224	60	732	7.1	3,256	131,436	112,865	152,666	9.0	
BB	1	6	1	47	0.2	12,680	474,511	391,615	565,634	32.7	
Hospital size											
Small	7	589	228	1,441	5.7	6,476	507,349	363,664	690,642	19.1	0.0061
Medium	15	690	257	1,727	6.7	7,430	406,425	275,406	584,308	15.3	
Large	69	3,988	1,511	7,198	38.7	13,608	758,313	404,071	1,251,025	28.5	
Very large	313	4,937	2,426	7,553	48.0	58,525	958,628	643,589	1,326,522	36.1	
Children's	16	92	38	222	0.9	4,681	27,646	18,077	42,534	1.0	
Sex											
Male	379	9,332	8,950	9,612	90.8	78,802	2,295,005	2,267,481	2,320,634	86.4	0.0033
Female	39	944	664	1,326	9.2	11,888	362,615	336,986	390,139	13.6	
Race											
White	80	2,830	2,241	3,481	35.1	23,843	931,455	740,477	1,133,254	42.6	0.059
Black	146	3,053	1,803	4,540	37.9	38,409	872,340	626,255	1,144,195	39.9	
Amerindian	64	2,072	1,193	3,287	25.7	9,368	363,148	203,062	610,719	16.6	
Asian	6	98	35	271	1.2	866	21,228	11,378	39,606	1.0	
Incident locale											
Home	68	1,969	1,510	2,461	41.6	20,327	732,937	631,178	836,432	47.1	0.011
School/recreation	14	354	153	775	7.5	1,986	78,502	64,005	96,242	5.0	
Street/highway	53	1,265	848	1,792	26.7	18,310	434,890	316,601	576,828	27.9	
Other property	47	1,144	834	1,523	24.2	10,250	304,040	252,129	363,632	19.5	
Farm	0	0	0	0	0.0	127	6,942	4,360	11,057	0.4	
Transportation to ED											
Emergency medical service	349	8,414	7,860	8,843	84.4	49,921	1,255,959	1,049,318	1,458,588	52.8	0.0001
Air	27	808	473	1,346	8.1	1,917	58,535	34,462	98,396	2.5	
Private vehicle	13	521	272	976	5.2	17,930	698,677	545,218	874,630	29.4	
Walk-in	5	144	64	321	1.4	8,161	283,043	203,446	388,355	11.9	
Police	5	79	15	418	0.8	2,977	72,219	35,413	145,217	3.0	
Other	0	0	0	0	0.0	237	8,278	4,040	16,399	0.3	
Anatomic location of injury											
Head/neck	126	3,015	3,593	306,716	29.6	25,521	793,383	740,891	847,663	30.5	0.0004
Upper trunk	138	3,605	4,305	366,737	35.4	14,266	381,696	334,378	434,639	14.7	
Lower trunk	148	3,427	3,894	348,629	33.7	11,123	289,804	257,815	325,003	11.1	
Upper extremity	1	82	610	8,342	0.8	14,783	491,967	442,972	545,057	18.9	
Lower extremity	4	44	137	4,476	0.4	23,075	647,338	615,109	680,735	24.9	
Diagnosis											
Contusion/abrasion	0	0	0	0	0.0	5,069	166,538	142,625	194,201	6.3	<10-4
Foreign body	23	576	1,222	59,305	5.6	9,135	323,836	248,409	417,875	12.3	
Fracture	163	3,752	4,747	386,306	36.4	6,523	188,859	157,624	225,779	7.2	
Laceration	34	839	1,410	86,383	8.1	9,807	335,217	263,935	422,348	12.7	
Internal organ injury	61	1,093	2,413	112,535	10.6	4,640	127,629	99,469	163,413	4.9	
Puncture	54	2,974	4,282	306,203	28.9	28,777	840,801	671,810	1,029,951	32.0	
Not stated	45	1,062	1,964	109,344	10.3	26,026	648,574	497,345	827,855	24.6	
ED Disposition											
Treated and released	35	1,309	793	2,086	12.8	51,326	1,686,619	1,503,591	1,855,563	64.0	0.0006
Admit	382	8,873	8,097	9,394	86.8	33,742	813,584	664,132	979,984	30.9	
Fatal	2	37	9	145	0.4	4,910	136,289	115,742	160,299	5.2	
Marital Status											
Never married	150	4,017	3,488	4,442	74.0	33,207	927,325	810,059	1,029,794	68.0	0.20
Married	26	910	585	1,364	16.8	7,619	308,953	244,696	384,366	22.7	
Divorced/separated	7	173	96	308	3.2	1,564	60,117	44,329	81,156	4.4	
Other	6	327	87	1,093	6.0	1,239	67,661	24,961	173,906	5.0	
Argument											
Yes	30	515	193	1,126	19.6	5,440	165,558	136,973	198,871	15.0	0.54
No	69	2,115	1,504	2,437	80.4	25,064	941,741	908,428	970,326	85.0	
Crime											
Yes	61	1,319	908	1,775	40.6	15,238	352,085	230,102	507,398	27.6	0.046
No	56	1,926	1,470	2,337	59.4	13,695	923,426	768,113	1,045,409	72.4	
Illicit drug involvement											
Yes	35	958	709	1,242	34.7	2,457	83,146	46,498	144,475	8.0	0.0052
No	60	1,804	1,520	2,053	65.3	25,781	954,749	893,420	991,397	92.0	
Fight											
Yes	36	872	555	1,291	25.1	7,768	229,151	189,007	275,510	19.0	0.20
No	83	2,609	2,190	2,926	74.9	25,879	973,951	927,592	1,014,095	81.0	
Alcohol involvement											
Yes	29	818	521	1,261	7.9	4,081	143,694	87,538	236,836	5.4	0.056
No	391	9,478	9,035	9,775	92.1	86,636	2,514,519	2,448,377	2,597,675	93.6	
Sexual assault											
Yes	0	0	0	0	0.0	505	13,067	2,640,018	2,649,057	0.5	0.0012
No	420	10,296	7,205	14,944	100.0	90,214	2,645,278	0	0	99.5	
Who caused											
Unknown	260	5,773	4,879	6,578	56.3	44,887	1,103,518	971,206	1,227,885	41.7	0.028
Stranger	54	1,400	1,083	1,776	13.7	13,311	369,553	314,307	428,240	14.0	
Self	30	1,017	627	1,596	9.9	17,077	683,502	558,034	817,357	25.9	
Friend/acquaintance	19	520	281	935	5.1	5,425	180,846	152,792	211,476	6.8	
Spouse/ex	2	59	12	281	0.6	522	19,137	15,332	23,527	0.7	
Other relative	5	135	60	294	1.3	2,065	76,853	61,593	94,636	2.9	
Other/not seen	50	1,392	1,008	1,875	13.6	7,433	224,952	184,778	270,161	8.5	

Patients with spinal injury

**Figure 2:**
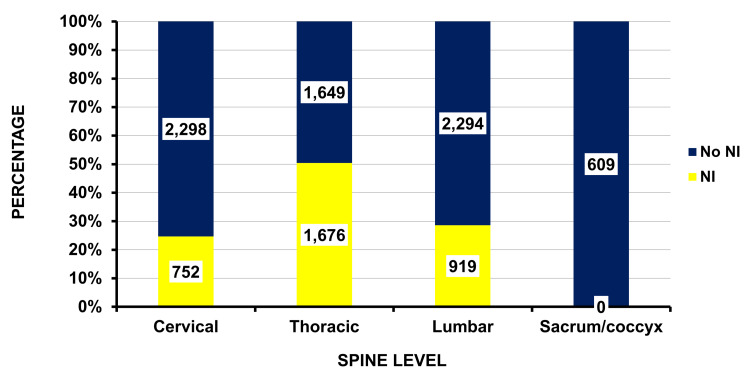
Differences by spine level with or without neurologic injury (NI) (p = 0.0003 includes sacrum/coccyx, and p = 0.01 excludes the sacrum/coccyx). The estimated numbers are given in each cell.

The anatomic location within the spine was identified in 10,197 (99.0%) of the injuries. The spinal level was 32% thoracic (3,325), 32% lumbar (3,213), 30% cervical (3,050), and 6% sacrococcygeal (609). The majority (91.8%) (9,438 - {8,863 - 9,793}) of the patients sustained a fracture. There were no differences between patients with and without a fracture by any of the variables in Table 1 or by spine level. We also compared those with and without a neurologic injury. Patients without a fracture were more likely to have sustained a neurologic injury (97.9 vs. 79.1% - p = 0.0037), and there was a significant difference in neurologic injury by spine level (Figure 2). Patients with injuries to the thoracic area had the highest percentage of neurologic involvement (50.4%), followed by the lumbar spine (28.6%) and the cervical spine (24.7%).

Non-powder firearm gunshot wound injuries

Although the majority (97.0%) of patients with spinal injuries associated with firearms involved a powder firearm gunshot wound, 2.9% involved a powder firearm without a gunshot wound. There was one case involving an air-powered firearm, indicating that air-powered weapons can also result in injury. To further explore this issue, the narrative comments of the actual (not estimated) 420 spine injury cases were reviewed to obtain an idea of the types of powder firearm non-gunshot wound injuries. There were 16 actual cases involving powder firearms without a gunshot wound. Four of these were due to falls from hunting stands resulting in spine fractures. The others were due to various assaults resulting in various injuries such as “the patient was assaulted with the handle of a 38-caliber handgun resulting in a closed head injury and C1 fracture.” Another example is “the patient was assaulted by multiple people and pistol-whipped, resulting in L2, 3, 4 fractures, and hemopneumothorax with rib fractures.” The single air-powered firearm wound occurred when a 12-year-old child was shot in the posterior thoracic area by his brother with a pellet gun, with the pellet lodged in the T11 neural foramen.

Changes over time

**Figure 3:**
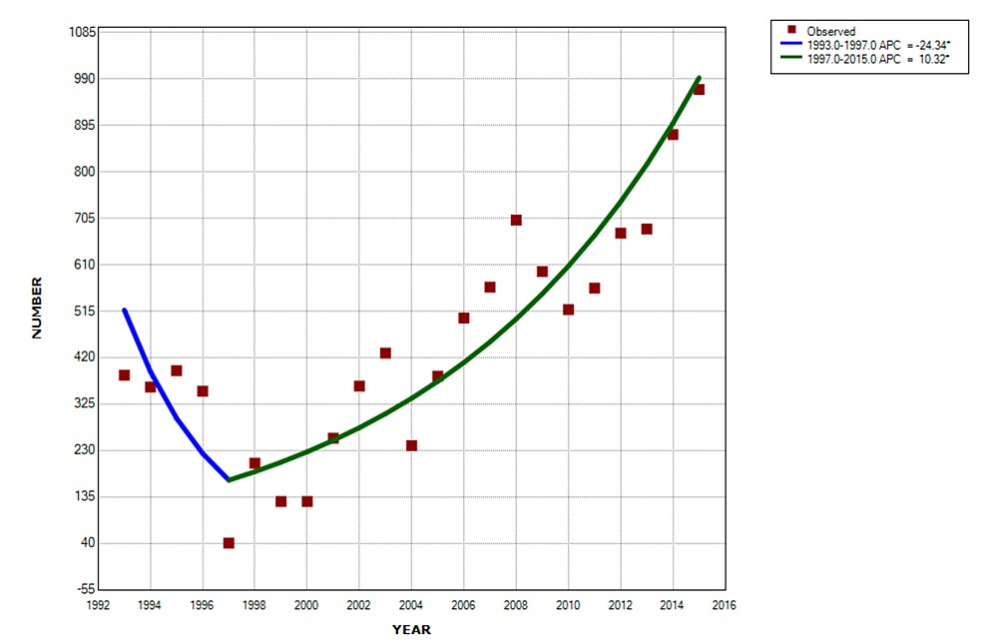
Joinpoint regression analyses or those with a spine injury There was an annual decrease of 24.3% from 1993 through 1997 (p = 0.016), and then an annual increase of 10.3% from 1997 through 2015 (p < 10-6).

**Figure 4:**
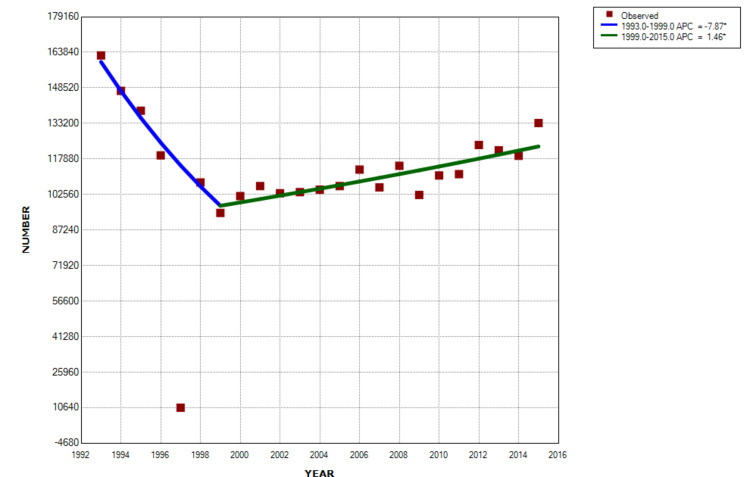
Joinpoint analyses for those without a spine injury There was an average annual decrease of 7.9% from 1993 to 1999 (p = 0.0002), and then an average annual increase of 1.5% from 1999 through 2015 (p = 0.0001).

Joinpoint regression demonstrated an annual percentage decrease for both those patients with and without spine involvement in the 1990s, followed by increases through 2015. The average percentage increase for patients with a spine injury was 10.3% per year from 1997 onwards (p < 10-6) (Figure 3), significantly greater than the 1.5% for those without spinal involvement (p = 0.0001) from 1999 onward (Figure 4).

## Discussion

**Figure 5:**
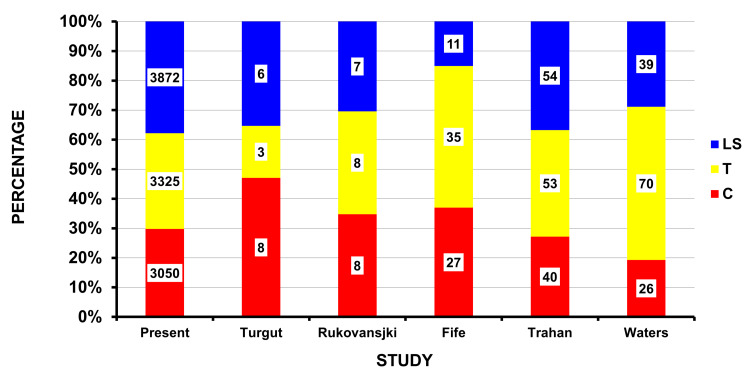
Location of spine injury due to firearms: present study and those in the literature The actual number of cases is shown in each cell.

There are few studies that allow us to compare the results of our present study. A compilation of the literature regarding civilian firearm injuries to the spine finds similar findings to those in this study (Table 2). Excluding those studies of only children, the average age was similar: 28 years in this study and 25 to 27 in the others [9-12]. The vast majority of the patients were male: 91% in this study and 80% to 94% in the literature [9-13]. The anatomic location of the injury was also similar (Figure 5).

**Table 2:** Literature comparison of spinal injuries due to firearms N = no, Y = yes, GSW = gunshot injury, SCI = spinal cord injury * the n is for only those with GSW s in each study ^ only children $ only those with spinal cord injuries; the others include both those with and without spinal cord injuries

	Present Study	Turgut [9]$	Rukovansjki [14]	Carillo [13]^	de Amoreira Gepp [15]^$	Fife [16]$	Rhee [10]	Trahan [11]	Waters [12]$
n*	10,296	17	20	19	11	73	168	147	135
GSW alone	N	Y	Y	Y	Y	N	N	Y	Y
SCI alone	N	Y	Y	Y	Y	Y	N	N	Y
Geographic location	All USA	Turkey	Croatia	Miami	Brazil	California	LA, Wash DC	New Orleans	California
Years studied	1993-2015	1968-1990	1991-1993	1992-1995	1996-2009	1970-1971	1993-2000	2007-2011	NA
Age (yrs)									
Average	28	25		17		-	26	27	25
Range	<1 to 112	16-40	12 to 57	14-19	0-10	-		14-66	17-59
% Male	91	82	80	95	-	-	92	92	94
Injury intent									
Unintentional	8	6		-	-	-	-	-	-
Assault	84	82	100	-	-	-	-	-	-
Self	6	12		-	-	-	-	-	-
Spine level (%)									
Cervical	30	47	40	16	18	37	100	27	19
Thoracic	32	18	40	21	73	48	0	36	52
Lumbosacral	38	35	35	63	9	15	0	36	29
Race (%)									
White	35.1	-	-	-	-	-	-	9.0	4.4
Black	37.9	-	-	-	-	-	-	84.0	46.7
Amerindian	25.7	-	-	-	-	-	-		45.2
Asian	1.2	-	-	-	-	-	-		
Drug involvement	35	-	-	37	-	-	-	39	
Alcohol involvement	0	-	-	26	-	-	-	16	

The vast majority (86.7%) of the patients with a spine injury were seen in large or very large hospitals (Table 1). This pattern likely indicates firearm injury due to urban violence [17-21], supported by the fact that 83.7% of the patients with a spine injury were injured during an assault. Although the number of spinal firearm injuries initially decreased in the 1990s, there was an annual 10.3% increase from 1997 through 2015. This likely reflects the epidemic of increasing firearm violence [4,22-23].

Non-powder weapons can result in serious injury [24-26], especially in children and adolescents. These injuries include blindness and paralysis [25], subarachnoid hemorrhage; lung, liver, and kidney lacerations; pulmonary artery injury; and tracheal injury [24], with 30% requiring an operative procedure [26]. One case in this study involved a 12-year-old child having a pellet gun missile becoming lodged in the T11 neural foramen, which is a significant injury and required hospital admission.

A neurologic injury was most frequent when the firearm injury involved the thoracic spine. This is likely due to the fact that cervical spine injuries, especially those involving the upper cervical spine (e.g. C1-4) can easily result in immediate/rapid death. It is possible that such a patient was never taken to an ED but was rather pronounced dead at the scene and transferred to the morgue. The proportion of spinal injuries between the cervical, thoracic, and lumbar areas was very similar. This is surprising because the available anatomic height differs among the different spinal regions, with the cervical spine having a smaller height than the thoracic or lumbar spine. The reason that the cervical spine had relatively equal numbers is unknown. One hypothesis is that perhaps the perpetrator was firing towards the head, but the bullet hit the cervical spine instead.

The demographics of firearm injuries point to potential prevention strategies for such injuries. In this study, 90.8% involved males, 83.7% an assault, 83.5% a handgun, 73.2% were 15 to 34 years, with many also involving a crime (40.6%) or drug activity (34.7%). Focusing interventions on these high-risk demographic groups is one prevention approach. Handgun control has certain efficacy [27] but is presently a very politically charged issue in the United States; how gun control laws may change in the future is unknown. Also, illegal handgun use is difficult to control [28]. In Philadelphia, reclaiming blighted vacant urban land significantly reduced shootings that resulted in serious injury or death between the years 2013 to 2015 [19]. Events involving a crime or drug activity are likely codependent; reducing illicit drug activity would hopefully result in less criminal activity as well.

The limitations of this study must be acknowledged. First is the accuracy of the NEISS data. However, previous studies [29-30], including those involving firearms, have demonstrated an over 90% accuracy of NEISS data. Next, the NEISS only identifies individuals who sought care in an ED. It does not include those who might have been treated in urgent care centers, physician offices, other venues, or those who did not seek any medical care. However, any person sustaining a spinal injury due to a firearm would likely present to an ED. Thus, the data presented in this study are likely very accurate. The NEISS does not allow for analyses by the socioeconomic status of the injured patient, nor detailed geographic regions (i.e. exactly which city and where in a particular city) but does allow for analyses by hospital size, which is a proxy of rural versus urban locations. Finally, the NEISS does not give details regarding treatment and outcomes except for disposition from the ED (release, admit, death). Acknowledging these limitations, the data led to the many interesting results noted above.

## Conclusions

The vast majority (98.2%) of spine injuries from firearms were due to powder firearm gunshot wounds. The average age was 28 years with very few < 14 years of age. The cervical spine was involved in 30%, thoracic in 32%, lumbar in 32%, and sacrum in 6%. A fracture occurred in 91.8% and neurologic injury in 33%. Injuries to the thoracic spine had the highest percentage of neurologic involvement (50.4%). This very large US-wide study of spinal injuries associated with firearms covering all ages can be used as baseline data for future firearm studies. The need for firearm injury research has been recently noted. A reduction in the incidence of such injuries can be guided by our findings, although it may be difficult. The relentless rise of 10.3% per year in firearm spine injuries is certainly a cause for concern.
